# Supplementary material for: Multiomics characteristics and immunotherapeutic potential of EZH2 in pan-cancer
Source: Biosci Rep. 2023 Jan 16;43(1):BSR20222230. doi: 10.1042/BSR20222230 (PMC9842950; doi:10.1042/BSR20222230)
Supplement: Supplementary Figures S1-S6 [file BSR-2022-2230_supp.pdf]

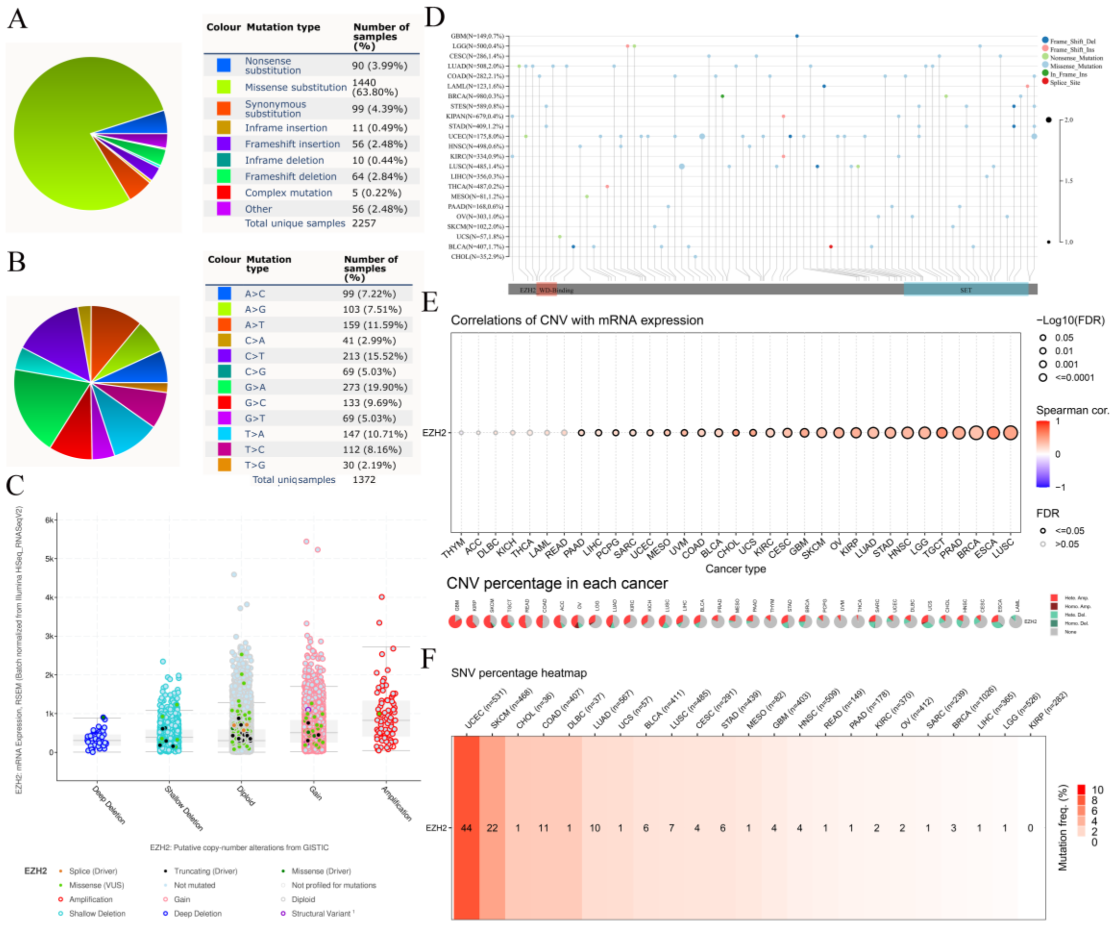

Supplementary file 1: Figure S1. **Mutation analysis of EZH2.** (**A**) the main mutation type of EZH2. (**B**) The primary single nucleotide variation (SNV) class type of EZH2. (**C**) Correlation between EZH2 expression and different mutated types. (**D**) The mutation spectrum of EZH2. (**E**) Association between EZH2 expression and copy number variation (CNV). (**F**) Correlation between EZH2 expression and SNV.

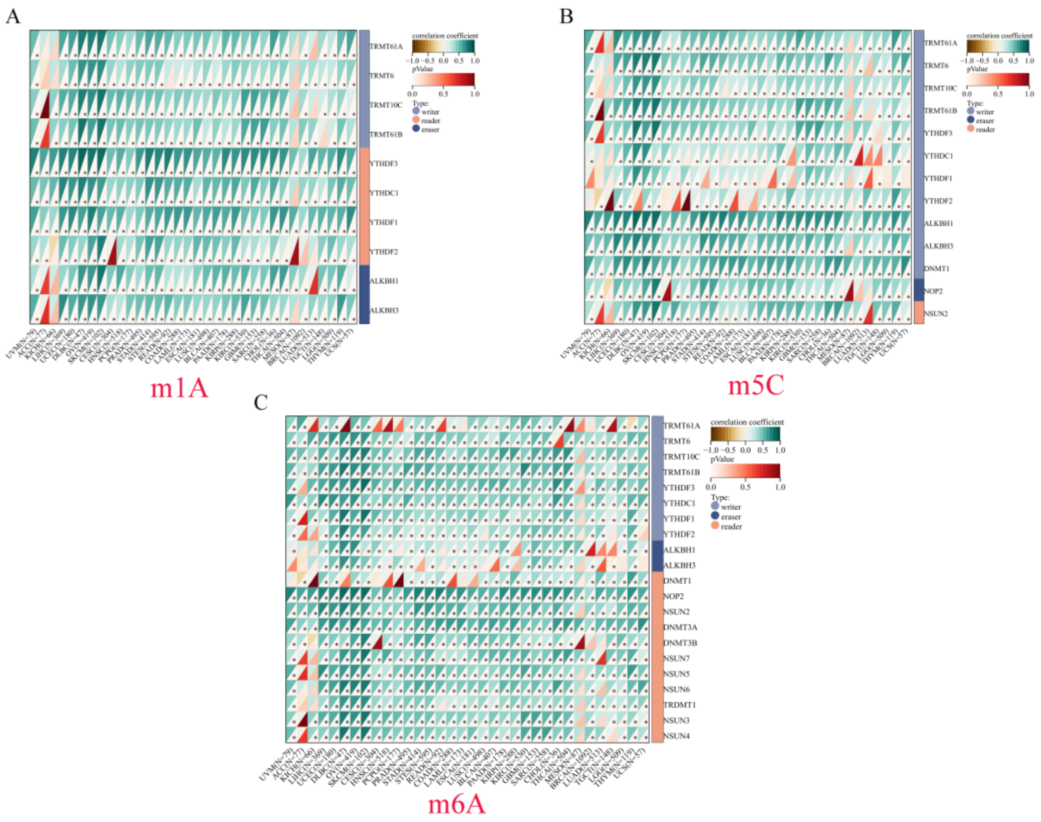

Supplementary file 2: Figure S2. **Correlation between EZH2 expression and RNA methylation regulators (i.e., m1A, m5C, m6A).** (**A**) m1A. (**B**) m5C. (**C**) m6A.

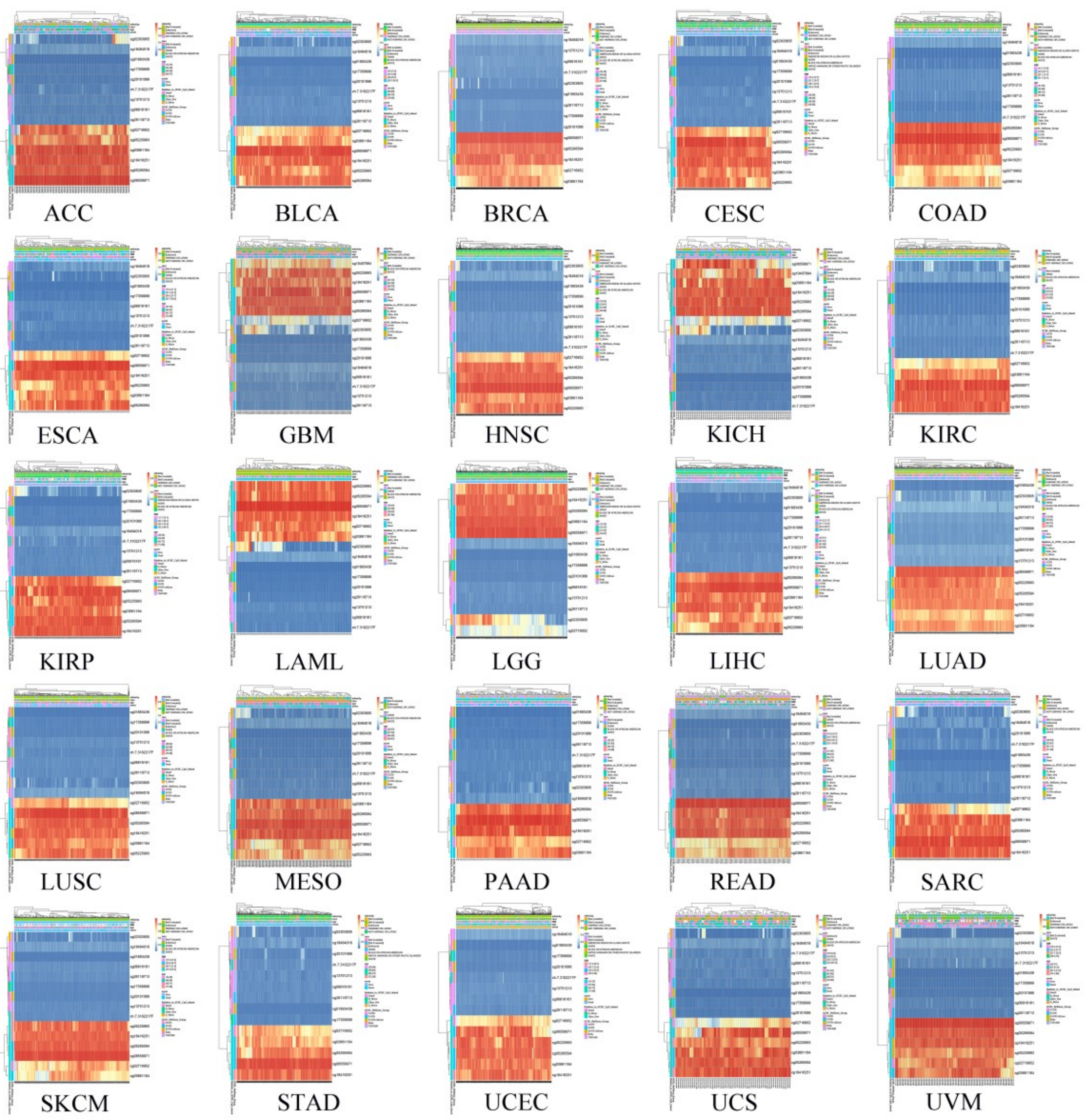

Supplementary file 3: Figure S3. **Heatmap of DNA methylation level of EZH2 in various cancers.**

High-expression (red); low-expression (bule).

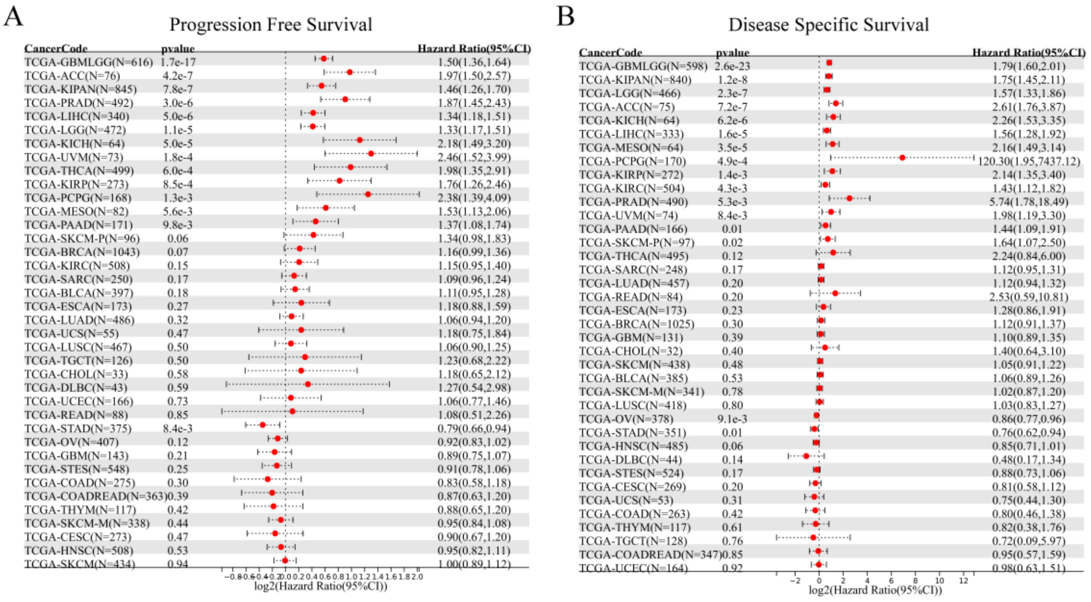

Supplementary file 4: Figure S4. **Correlation between EZH2 expression and survival prognosis of TCGA pan-cancer patients.** (**A**) Progression free survival. (**B**) Disease specific survival.

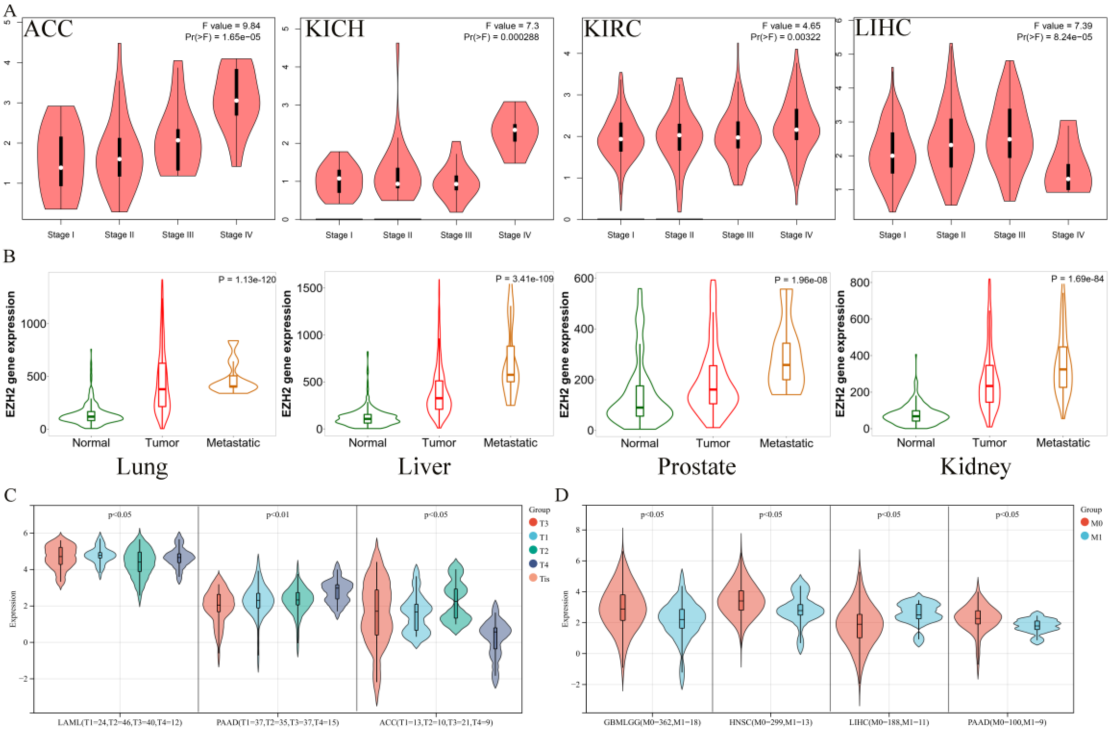

Supplementary file 5: Figure S5. **Association analysis of EZH2 expression with clinicopathological features.** **(A)** The association between EZH2 expression and pathological stages. (**B**) Violin plots showing differential EZH2 expression levels in different metastasis. (**C-D**) Correlation between EZH2 expression and TNM classification (T and M stages).

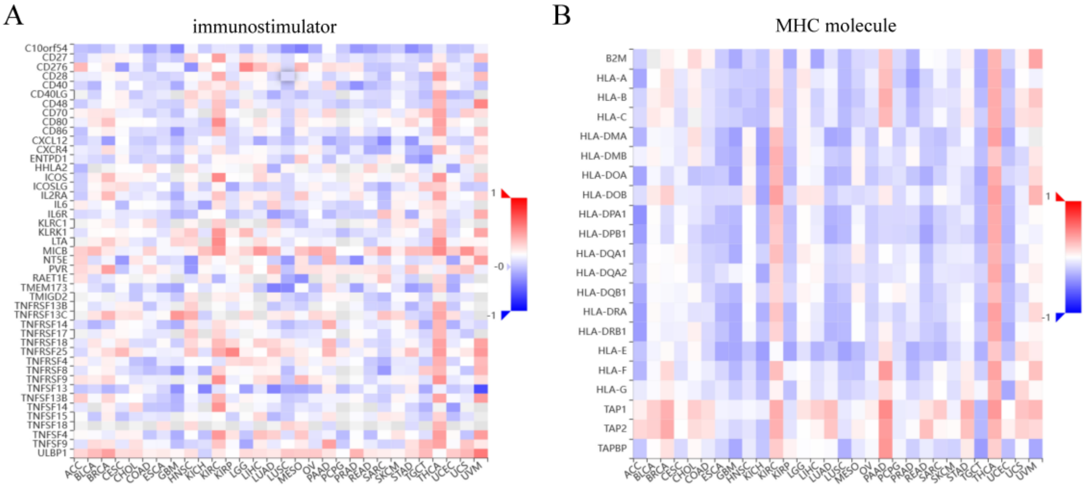

Supplementary file 6: Table S6. **The correlation between EZH2 expression and several immunotherapy-related molecules.** (**A**) Immunostimulator. (**B**) MHC.
