# Supplementary material for: Multiomics characteristics and immunotherapeutic potential of EZH2 in pan-cancer
Source: Biosci Rep. 2023 Jan 16;43(1):BSR20222230. doi: 10.1042/BSR20222230 (PMC9842950; doi:10.1042/BSR20222230)
Supplement: Supplementary Tables S1-S2 [file BSR-2022-2230_supp1.zip › Supplementary ftable-caption.pdf]

Supplementary file 7: Table S1. **The result of predicted miRNAs based on miRNet database**

Supplementary file 8: Table S2. **The result of predicted miRNAs on the basis of Starbase database**
